# Prevalence and Burden of Breathlessness in Patients with Chronic Obstructive Pulmonary Disease Managed in Primary Care

**DOI:** 10.1371/journal.pone.0085540

**Published:** 2014-01-10

**Authors:** Hana Müllerová, Chao Lu, Hao Li, Maggie Tabberer

**Affiliations:** 1 Respiratory Epidemiology, GlaxoSmithKline R&D, Uxbridge, United Kingdom; 2 Observational Data Analytics, GlaxoSmithKline R&D, Research Triangle Park, North Carolina, United States of America; 3 Global Health Outcomes, GlaxoSmithKline R&D, Uxbridge, United Kingdom; Clinica Universidad de Navarra, Spain

## Abstract

**Background & Aims:**

Breathlessness is a primary clinical feature of chronic obstructive pulmonary disease (COPD). We aimed to describe the frequency of and factors associated with breathlessness in a cohort of COPD patients identified from the Clinical Practice Research Datalink (CPRD), a general practice electronic medical records database.

**Methods:**

Patients with a record of COPD diagnosis after January 1 2008 were identified in the CPRD. Breathlessness was assessed using the Medical Research Council (MRC) dyspnoea scale, with scoring ranging from 1–5, which has been routinely administered as a part of the regular assessment of patients with COPD in the general practice since April 2009. Stepwise multivariate logistic regression estimated independent associations with dyspnoea. Negative binomial regression evaluated a relationship between breathlessness and exacerbation rate during follow-up.

**Results:**

The total cohort comprised 49,438 patients diagnosed with COPD; 40,425 (82%) had any MRC dyspnoea grade recorded. Of those, 22,770 (46%) had moderate-to-severe dyspnoea (MRC≥3). Breathlessness increased with increasing airflow limitation; however, moderate-to-severe dyspnoea was also observed in 32% of patients with mild airflow obstruction. Other factors associated with increased dyspnoea grade included female gender, older age (≥70 years), obesity (BMI ≥30), history of moderate-to-severe COPD exacerbations, and frequent visits to the general practitioner. Patients with worse breathlessness were at higher risk of COPD exacerbations during follow-up.

**Conclusions:**

Moderate-to-severe dyspnoea was reported by >40% of patients diagnosed with COPD in primary care. Presence of dyspnoea, including even a perception of mild dyspnoea (MRC = 2), was associated with increased disease severity and a higher risk of COPD exacerbations during follow-up.

## Introduction

Dyspnoea or breathlessness is a primary clinical feature of chronic obstructive pulmonary disease (COPD) [1], [2]. Dyspnoea does not have a well-defined or universally accepted definition [3]. The American Thoracic Society defines dyspnoea as “the subjective experience of breathing discomfort that consists of qualitatively distinct sensations that vary in intensity” [4].

Dyspnoea is a distressing symptom, originating from a complex of physiological mechanisms [5], that significantly contributes to disease burden and poor quality of life [6], [7]. Despite the wide range of available treatments, as many as 50% of patients with COPD continue to experience significant dyspnoea with daily activities [8]–[12]. Hyperinflation associated with exertional dyspnoea is thought to develop early in the disease process [1]. Patient-reported dyspnoea progresses despite stable lung function [13] and has been associated with mortality in a severity-dependent manner [14].

Multiple methods for assessing dyspnoea exist, but most have not found a place in daily clinical practice [15]. An exception is the Medical Research Council (MRC) dyspnoea scale, a unidimensional measure of breathlessness related to activities [16] (table 1). The MRC dyspnoea scale (1–5) is a simple measure of breathlessness associated with exercise [2] and has been used in many studies (for review see reference [17]). The more widely used version, the modified MRC dyspnoea grade, only differs on the grading (0–4).

**Table 1:** Medical Research Council dyspnoea grades (16).

Grade	Degree of breathlessness related to activities
1	Not troubled by breathlessness except on strenuous exercise
2	Short of breath when hurrying or walking up a slight hill
3	Walks slower than contemporaries on level ground because of breathlessness, or has to stop for breath when walking at own pace
4	Stops for breath after walking about 100 m or after a few minutes on level ground
5	Too breathless to leave the house, or breathless when dressing or undressing

Adapted with permission from Fletcher CM, Elmes PC, Fairbairn MB, et al. The significance of respiratory symptoms and the diagnosis of chronic bronchitis in a working population. *Br Med J* 1959; 2∶257–266, as used in the UK National Health Service Quality and Outcomes Framework for COPD (16).

Several cross-sectional studies have reported the high prevalence of dyspnoea in populational samples of respondents self-reporting diagnosis of COPD or chronic bronchitis (∼70–80% with MRC≥1) [18], [19] or with airflow limitation identified using screening with spirometry (49%) [20]. However, there is limited information about the occurrence, distribution and outcomes associated with dyspnoea among patients with diagnosed COPD who are managed in primary care. A cross-sectional study of COPD patients selected from primary care offices in several European countries [9] reported an 80% prevalence of dyspnoea MRC≥1; however, these date are from a selective group of patients and it was not possible to show an association with prospectively evaluated outcomes.

To our knowledge, recording of dyspnoea in electronic medical record (EMR) databases has not been reported in patients with COPD. Claims databases created for health insurance billing purposes usually omit symptom recording. EMR databases often employ universally accepted classifications, such as International Classification of Disease codes, but these do not provide standardized methods of symptom recording. Exceptions occur when national guidelines require the collection of such information or where it is collected for specific studies and made more widely available to the research community.

Since April 2004, COPD indicators have been included in the UK National Health Service Quality and Outcomes Framework (QOF) as a part of the General Medical Services contract for general practitioners (GPs). QOF is a voluntary scheme incentivising GP practices to provide high-quality care that is recorded in a standardized reporting system; practices are awarded points according to performance indicators. Since April 2009, the MRC dyspnoea grade has been routinely collected as an indicator in the annual review of patients with COPD [21]–[23].

The aims of the current study were to (1) describe the distribution of MRC dyspnoea grades in a primary care COPD population, (2) evaluate factors associated with reporting of increased dyspnoea in this population, and (3) evaluate any relationships between dyspnoea and future exacerbation frequency over 12 months.

## Methods

### COPD Cohort Selection

A COPD cohort was identified in the Clinical Practice Research Datalink (CPRD), an anonymized collection of EMR from general practices in England and Wales managed by the Medicines and Healthcare products Regulatory Agency (http://www.cprd.com/intro.asp). The study protocol (WEUSKOP5224) was approved by the Clinical Practice Research Datalink Scientific and Ethics committee.

Patients diagnosed with COPD, confirmed with spirometry (forced expiratory volume in 1 second [FEV_1_]/forced vital capacity<70%), after January 1 2008 (date of COPD medical diagnosis = cohort entry) were identified. FEV_1_ and the FEV_1_/FVC ratio are routinely recorded in the EMR of COPD patients in the UK as a part of QOF [21], [22], [24]. The QOF specification recommends general practices report the percentage of all patients with a diagnosis of COPD that has been confirmed by post-bronchodilator spirometry. The CPRD contains records of the actual values recorded for both FEV_1_ and the FEV_1_/FVC ratio, and less frequently, of other spirometric values. Due to the retrospective nature of this study, the quality control of spirometry could not be reviewed.

The study period was from April 1 2009 (or cohort entry date if this was later) and finished at censoring (death, left practice or end of follow-up as of March 31 2011). All patients were required to have a minimum of 24 months’ COPD history: 12 months before and 12 months after the start of the study period. This COPD patient population represents prevalent COPD patients who consulted with their GP at the start of cohort entry. The first recorded MRC dyspnoea grade during the observation period was used to ascertain dyspnoea.

A subset of patients from this cohort was created for the analysis of factors associated with dyspnoea. Patients were required to have a minimum of 12 months’ EMR data available before and after the first MRC dyspnoea grade recorded after April 1 2009 or cohort entry date if this was later (i.e. no censoring was allowed for the 12 months following cohort entry). The study period for this analysis was from the date of the first MRC dyspnoea grade; the covariates were derived in relationship to this re-defined observation period start.

### Dyspnoea Assessment

Dyspnoea was assessed using recorded MRC dyspnoea grade routinely collected in general practice as a COPD disease indicator (table 1) [16]. The indicator, QOF COPD 13, requires participating practices to record *“The percentage of patients with COPD who have had a review, undertaken by a healthcare professional, including an assessment of breathlessness using the MRC dyspnoea grade in the preceding 15 months”* [21]. Dyspnoea grade should be collected during an annual COPD review, when a patient is presumably in stable state. However, due to the nature of this analysis, using retrospective non-interventional data from an electronic medical records database, we were not able to standardize the administration of the scale.

MRC dyspnoea grade 1 is defined as no breathlessness, grade 2 as mild dyspnoea and ≥grade 3 as, in agreement with published literature [2], moderate-to-severe or clinically significant dyspnoea. The more widely used version, the modified MRC (mMRC) dyspnoea grade, only differs on the grading (0–4) with MRC grade 1 equal to mMRC grade of 0.

A feasibility study was conducted to provide information on the use of MRC grades to record information on dyspnoea in this database.

### Other Assessments

Information was also extracted for age, gender, smoking status, body mass index (BMI; or imputed using the latest height and weight measurements where BMI data were not recorded) and FEV_1_ percent predicted assessment with the closest date to observation period start. COPD severity was categorized according to the Global Initiative for Obstructive Lung Disease (GOLD) 2006 stage classification based on the level of airflow obstruction [25]. Due to the nature of data retrieved from the database, we were not able to ascertain whether only post-bronchodilator spirometry data were recorded. Hence, we created modified categories of airflow limitation severity, using cut points of FEV_1_≥80% predicted for mild (Stage I), ≥50% to <80% FEV_1_ predicted for moderate (Stage II), ≥30% to <50% FEV_1_ predicted for severe (Stage III), and >30% FEV_1_ predicted for very severe level of airflow limitation (Stage IV).

Records of comorbidities were retrieved for any time before the study start and were described (a) using the Charlson comorbidity index [26] and (b) by frequency of key individual comorbidities, including those listed in the comorbidity index and also depression, anxiety and asthma.

Data were extracted for frequency of prescription treatment for COPD from 12 months before study start. Patients with one or more record for a medication in a therapeutic class were considered users, except for oral corticosteroids for which four or more prescriptions within 12 months was considered regular use (as opposed to acute use for COPD exacerbations).

Data on the number of all general practice healthcare utilizations for any reason in the 12 months before study start was collected and standardized per 365.25 days. The total number of GP office visits was used as a covariate in multivariate models.

Data on moderate-to-severe COPD exacerbations from 12 months before and 12 months after study start were collected. Hospital admissions or emergency room visits for COPD were considered as severe exacerbations. Moderate exacerbations were defined as either a record of a diagnosis of exacerbation, or management with selected antibiotics and oral corticosteroids co-prescribed within 5 days.

### Statistical Analysis

Characteristics of COPD patients by dyspnoea grade were tabulated. Bivariate relationships between MRC dyspnoea grade were evaluated (a) using the Cochran-Mantel-Haenszel test (for categorical or ordinal variables), (b) using polyserial correlations (for continuous variables), or (c) using negative binomial modeling with exacerbation frequency as the predictor variable while controlling for the age and gender to assess a relationship with count data. The correlation between airflow limitation and MRC dyspnoea grade was evaluated using the Pearson correlation coefficient.

Independent associations with dyspnoea grades were assessed with stepwise multivariate multinomial logistic regression for the simultaneous comparisons of (a) MRC = 1 vs MRC = 2, (b) MRC = 1 vs MRC≥3, and (c) MRC = 2 vs MRC≥3 (PROC CATMOD); binary logistic regression for the secondary model (MRC = 1 or MRC = 2) vs MRC≥3 (PROC LOGISTIC) was used. Negative binomial modelling, adjusted for multiple characteristics (age, gender, BMI, GOLD stage, history of comorbidities, prior history of COPD exacerbations, prior contacts with GP), was used to evaluate a relationship between dyspnoea (MRC grades 1, 2 and ≥3) and COPD exacerbations during the 12 months’ follow-up. Similar models were further split by GOLD stage. Statistical analyses were performed using SAS software, version 9.1 (SAS Institute, Cary, North Carolina, USA). All statistical tests were 2-sided, and p<0.05 was considered statistically significant.

## Results

### Characteristics of COPD Patients with Dyspnoea

**Figure 1:**
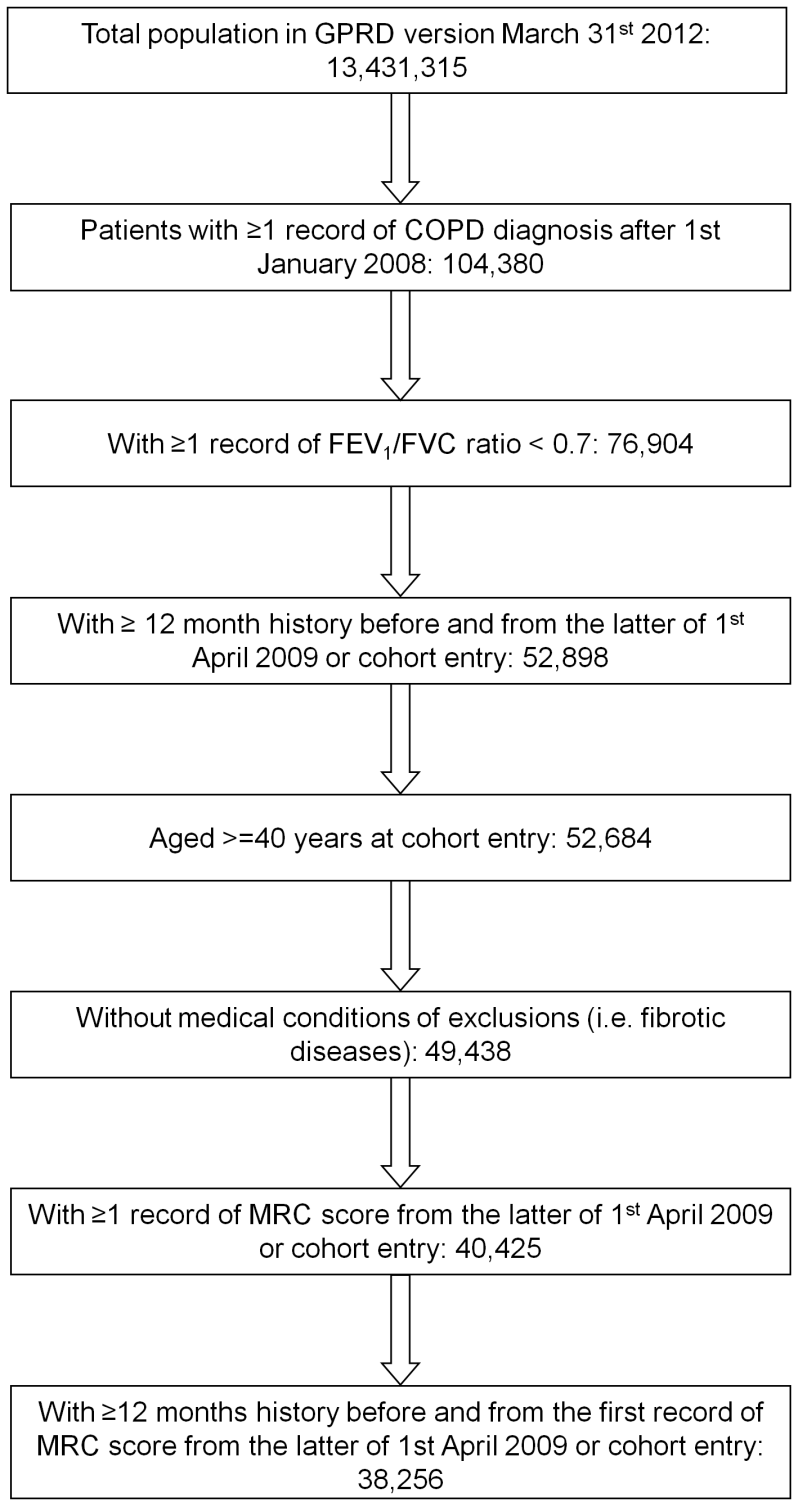
Cohort selection: flow diagram. Abbreviations: COPD, chronic obstructive pulmonary disease; FEV_1_/FVC, forced expiratory volume in 1 second/forced vital capacity; GPRD, General Practice Research Database; MRC, Medical Research Council.

The total cohort comprised 49,438 patients with COPD across all stages of airflow limitation severity (fig. 1); the mean (standard deviation) age was 69.2 (10.3) years, 46% were females and 33% were current smokers (table 2).

**Table 2:** Demographics and clinical characteristics of the total COPD cohort (at observation period start) further split by the MRC dyspnoea grade (first recorded from study start).

Variable	Total cohort	MRC grade (N = 40,425)					p-value across MRC grades
		1	2	3	4	5	
N	49,438	7392	15,378	10,529	5813	1313	
Females, n (%)	22,804 (46.1)	2915 (15.7)	7193 (38.8)	5035 (27.2)	2740 (14.8)	656 (3.5)	<0.001
Age, mean (SD)	69.2 (10.3)	66.4 (10.1)	68.4 (9.9)	70.1 (10.1)	71.3 (10.0)	73.2 (9.8)	<0.001
Current smoker, n (%)	16,341 (33.1)	2459 (18.3)	5249 (39.0)	3564 (26.5)	1817 (13.5)	379 (2.8)	0.377
Former smoker, n (%)	26,046 (52.7)	3795 (17.8)	7924 (37.2)	5510 (25.9)	3255 (15.3)	801 (3.8)	
Never smoker, n (%)	5066 (10.3)	855 (20.9)	1570 (38.4)	1032 (25.3)	531 (13.0)	98 (2.4)	
Other smoker, n (%)^a^	1985 (4.0)	283 (17.8)	635 (40.0)	423 (26.7)	210 (13.2)	35 (2.2)	
BMI <18.5, n (%)	2297 (4.7)	239 (13.4)	566 (31.8)	487 (27.4)	349 (19.6)	138 (7.8)	<0.001
BMI 18.5–24.9, n (%)	16,792 (34.0)	2689 (19.6)	5241 (38.2)	3438 (25.1)	1898 (13.8)	455 (3.3)	
BMI 25–29.9, n (%)	16,679 (33.7)	2880 (20.9)	5464 (39.6)	3437 (24.9)	1662 (12.1)	345 (2.5)	
BMI ≥30, n (%)	12,805 (25.9)	1467 (14.0)	3847 (36.7)	3012 (28.7)	1816 (17.3)	347 (3.3)	
Airflow limitation stage, n (%)							
Stage I (FEV_1_≥80% pred.)	7356 (14.9)	1640 (28.0)	2369 (40.5)	1281 (21.9)	479 (8.2)	81 (1.4)	<0.001
Stage II (≥50% FEV_1_<80% pred.)	24,815 (50.2)	4350 (21.0)	8673 (41.9)	5066 (24.5)	2276 (11.0)	337 (1.6)	
Stage III (≥30% FEV_1_<50% pred.)	13,126 (26.6)	1101 (10.3)	3569 (33.4)	3318 (31.0)	2196 (20.5)	503 (4.7)	
Stage IV (FEV_1_>30% pred.)	2911 (5.9)	110 (4.8)	445 (19.6)	641 (28.2)	726 (31.9)	352 (15.5)	
Moderate-to-severe COPD exacerbation in previous12 months, n (%) with at least one event	22,088 (44.7)	2448 (14.0)	6103 (34.8)	4949 (28.2)	3187 (18.2)	847 (4.8)	<0.001
Severe COPD exacerbation rate, n (%)with at least one event	5092 (10.3)	490 (12.5)	1185 (30.2)	1102 (28.0)	880 (22.4)	273 (6.9)	<0.001
GP visits, all contacts with GP surgery,mean (SD)	45.4 (27.4)	36.6 (22.4)	41.4 (24.1)	47.0 (26.9)	52.8 (29.0)	60.1 (30.9)	<0.001
GP visits, contacts with GP, mean (SD)	12.3 (9.4)	10.4 (8.0)	11.6 (8.5)	12.9 (9.6)	13.9 (10.2)	13.6 (10.2)	<0.001

Percentages are based on row total. MRC scoring 1–5 equals to mMRC grades 0–4 with MRC 1 equals to mMRC 0.

^a^ Other smoker: no record about smoking history or record of passive smoking.

Data on BMI were missing for 865 (1.7%) patients.

Data on GOLD grade were missing for 1002 (2%) patients.

Abbreviations: BMI, body mass index; COPD, chronic obstructive pulmonary disease; GOLD, global initiative for obstructive lung disease; GP, general practitioner; MRC, Medical Research Council; SD, standard deviation.

**Figure 2:**
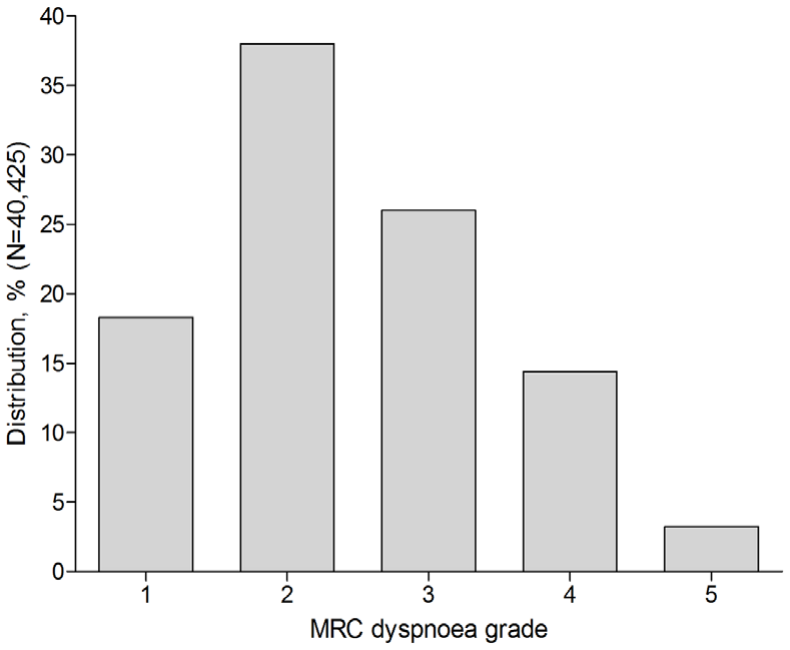
Distribution of MRC dyspnoea grade in the COPD cohort (first MRC grade from the study start). Abbreviations: COPD, chronic obstructive pulmonary disease; MRC, Medical Research Council. MRC scoring 1–5 equals to mMRC grades 0–4 with MRC 1 equals to mMRC 0.

Among 40,425 patients (82% of the total cohort) with at least one MRC dyspnoea grade recorded during the study period, mild dyspnoea (MRC = 2; equal to mMRC 1) was the most frequently reported single grade (38%); 44% of patients were classified as having moderate-to-severe dyspnoea (MRC≥3, equal to mMRC≥2) (fig. 2). Patients with MRC≥3, compared with those with mild dyspnoea (MRC = 2, equal to mMRC = 1) or those with absence of dyspnoea record (MRC = 1, equal to mMRC = 0), were older (mean age: 70.7 vs 67.8 years, p<0.001), more often female (48% vs 44%, p<0.001), had worse lung function (FEV_1_<50% predicted: 44% vs 23%, p<0.001), had more comorbidities, including heart failure (10% vs 5%, p<0.001) and were more intensively treated with COPD medications. Patients with MRC≥3 were also more frequently treated for COPD exacerbations in the past year (1.0 vs. 0.6 events per person per year, p<0.001). Patients with mild dyspnoea (MRC = 2) exhibited more disease burden and higher frequency of comorbidities compared with patients with absence of dyspnoea (MRC = 1) (see tables 2 and 3, also include significance testing for trends across dyspnoea grades).

**Table 3:** Comorbidities and respiratory medications in the total COPD cohort (at study start) further split by the MRC dyspnoea grade (first recorded from study start).

Variable	Total cohort	MRC grade (N = 40,425)					p-value across MRC grades
		1	2	3	4	5	
N	49,438	7392	15,378	10,529	5813	1313	
Charlson comorbidity index grade,mean (SD)	2.5 (1.6)	2.2 (1.4)	2.4 (1.5)	2.5 (1.6)	2.6 (1.7)	2.8 (1.7)	<0.001
Comorbidity, n (%)							
Acute MI	4558 (9.2)	436 (12.4)	1140 (32.5)	1093 (31.2)	666 (19.0)	170 (4.9)	<0.001
Heart failure	3932 (8.0)	257 (9.0)	811 (28.6)	875 (30.8)	687 (24.2)	210 (7.4)	<0.001
Stroke	2812 (5.7)	276 (12.2)	702 (31.0)	724 (32.0)	452 (20.0)	111 (4.9)	<0.001
Depression	6755 (13.7)	797 (14.9)	1943 (36.4)	1476 (27.7)	907 (17.0)	213 (4.0)	<0.001
Anxiety	8556 (17.3)	1094 (16.1)	2528 (37.2)	1871 (27.5)	1042 (15.3)	267 (3.9)	<0.001
Asthma	21,688 (43.9)	3088 (17.5)	6629 (37.5)	4660 (26.3)	2711 (15.3)	606 (3.4)	<0.001
Medication, n (%)							
ICS	10,451 (21.1)	1664 (19.2)	3454 (39.8)	2237 (25.7)	1127 (13.0)	207 (2.4)	<0.001
LABA	4823 (9.8)	532 (13.4)	1508 (37.9)	1170 (29.4)	642 (16.1)	130 (3.3)	0.169
ICS/LABA combination product	25,611 (51.8)	2769 (13.3)	7169 (34.5)	5912 (28.5)	3923 (18.9)	983 (4.7)	<0.001
LAMA	19,374 (39.2)	1610 (10.3)	5109 (32.7)	4753 (30.4)	3324 (21.3)	834 (5.3)	<0.001
Theophyllines	2976 (6.0)	179 (7.9)	533 (23.4)	627 (27.5)	692 (30.4)	248 (10.9)	<0.001
SABD	40,270 (81.5)	5130 (15.6)	12,311 (37.4)	9009 (27.3)	5281 (16.0)	1218 (3.7)	<0.001
Oral corticosteroid, long-term treatment^a^	3163 (6.4)	198 (8.6)	567 (24.6)	640 (27.8)	644 (28.0)	254 (11.0)	<0.001

Percentages are based on row total. Comorbidities were searched for any time in the history before the observation period start; respiratory medications were searched for 12 months prior to study start. MRC scoring 1–5 equals to mMRC grades 0–4 with MRC 1 equals to mMRC 0.

^a^≥4 treatments per year.

Abbreviations: COPD, chronic obstructive pulmonary disease; ICS, inhaled corticosteroids; LABA, long-acting beta-blockers; LAMA, long-acting anticholinergics; MI, myocardial infarction; MRC, Medical Research Council; SABD, short-acting bronchodilators; SD, standard deviation.

**Figure 3:**
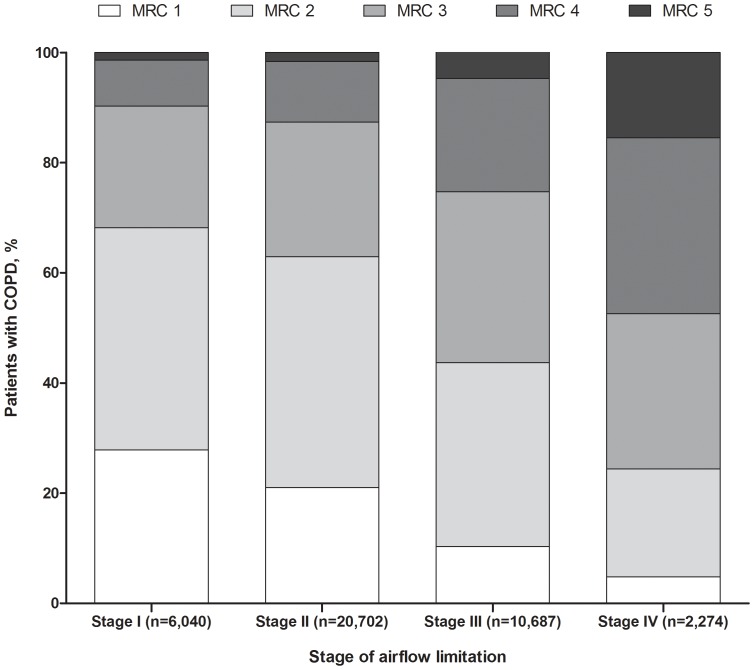
MRC dyspnoea grade distribution by stage of airflow limitation (first MRC grade from study start). Abbreviations: COPD, chronic obstructive pulmonary disease; MRC, Medical Research Council. MRC scoring 1–5 equals to mMRC grades 0–4 with MRC 1 equals to mMRC 0.

**Figure 4:**
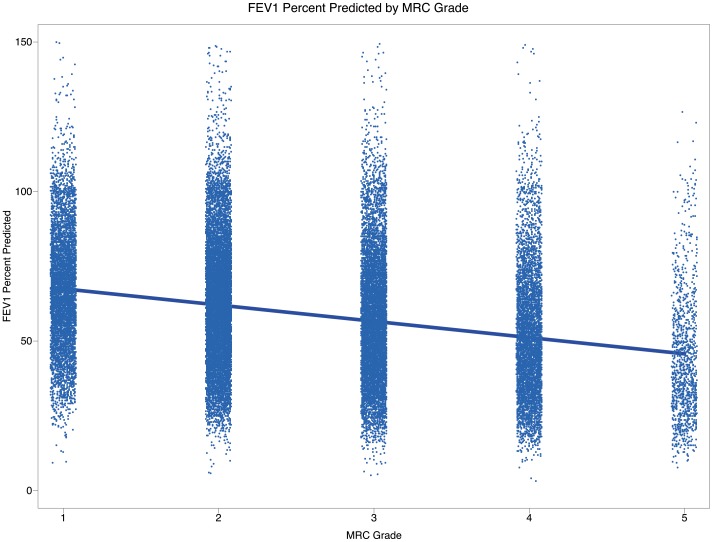
Bivariate relationship between FEV_1%_ predicted and MRC grade: scatter plot. Abbreviations: FEV_1%_predicted, forced expiratory volume in one second; Stage I: FEV_1_≥80% predicted; Stage II: ≥50% to <80% FEV_1_ predicted; Stage III: ≥30% to <50% FEV_1_ predicted; Stage IV: >30% FEV_1_ predicted; MRC, Medical Research Council. MRC scoring 1–5 equals to mMRC grades 0–4 with MRC 1 being equal to mMRC 0.

Dyspnoea severity increased with increasing severity of airflow limitation (fig. 3); however, the overall relationship was weak (Pearson r = –0.28) (fig. 4).

### Determinants and Outcomes Associated with Level of Dyspnoea

The determinants of MRC dyspnoea grade and its relationship with COPD exacerbations recorded during 12 months’ follow-up(following a record of MRC dyspnoea grade) was examined in a subgroup of patients with a minimum of 12 months’ history before and after the first recorded MRC dyspnoea grade. The subgroup comprised 38,256 patients with COPD with the same characteristics as the overall cohort.

Factors associated with the increasing level of dyspnoea are shown in table 4. The risk of higher dyspnoea grade increased most noticeably with increasing severity of airflow limitation. In addition, dyspnoea was increased in those with high BMI (BMI ≥30), females and with a past history of moderate-to-severe COPD exacerbations (odds ratio [OR], 95% confidence interval [CI]: 1.44, 1.37–1.51 for MRC≥3 vs MRC = 1 and 1.13, 1.07–1.18 for MRC = 2 vs MRC = 1). A higher risk of more severe dyspnoea was associated with most comorbid diagnoses. A diagnosis of heart failure presented with the most consistent pattern and highest risk across dyspnoea grades (OR, 95% CI: 1.32, 1.22–1.43 for MRC≥3 vs MRC = 1). Significant differences were observed between the three pre-defined comparisons, indicating separation between mild dyspnoea and no dyspnoea (MRC = 1 vs MRC = 2) and mild dyspnoea and moderate-to-severe dyspnoea (MRC = 2 vs MRC≥3).

**Table 4:** Factors associated with level of dyspnoea. MRC scoring 1–5 equals to mMRC grades 0–4 with MRC 1 being equal to mMRC 0.

Factor	Dyspnoea grade					
	MRC≥3 vs MRC = 1		MRC = 2 vs MRC = 1		MRC≥3 vs MRC = 2	
	Odds ratio	95% CI	Odds ratio	95% CI	Odds ratio	95% CI
Sex (male)	0.77	0.75–0.80	0.84	0.82–0.87	0.91	0.89–0.94
Age group, years						
40–49	0.51	0.44–0.58	0.69	0.61–0.78	0.74	0.65–0.83
50–59	Referent					
60–69	0.83	0.78–0.88	0.96	0.91–1.01	0.87	0.83–0.91
70–79	1.29	1.22–1.38	1.16	1.09–1.23	1.12	1.07–1.18
≥80	2.69	2.48–2.92	1.66	1.53–1.80	1.62	1.52–1.72
Smoking status						
Current smoker	Referent					
Ex-smoker	0.94	0.89–1.00	0.95	0.90–1.01	0.99	0.94–1.03
Non-smoker	0.71	0.65–0.77	0.77	0.71–0.84	0.92	0.86–0.99
Other^a^	1.15	1.02–1.30	1.16	1.04–1.31	0.99	0.90–1.09
Body mass index, kg/m^2^						
<18.5, underweight	0.96	0.85–1.09	0.90	0.79–1.02	1.08	0.99–1.18
18.5–24.9, normal	Referent					
25–29.9, overweight	0.80	0.75–0.85	0.92	0.87–0.98	0.87	0.83–0.91
≥30, obese	1.71	1.60–1.83	1.39	1.30–1.49	1.23	1.17–1.29
Airflow limitation level nearest toobservation period start						
Stage I (FEV_1_≥80% pred.)	Referent					
Stage II (≥50% FEV_1_<80% pred.)	0.43	0.40–0.46	0.74	0.69–0.80	0.58	0.55–0.60
Stage III (≥30% FEV_1_<50% pred.)	1.47	1.36–1.59	1.29	1.19–1.40	1.14	1.09–1.19
Stage IV (FEV_1_>30% pred.)	5.85	4.98–6.88	1.95	1.64–2.31	3.00	2.76–3.27
Comorbidities anytime beforethe observation period start						
Diabetes	1.06	1.01–1.12	0.99	0.94–1.04	1.08	1.04–1.12
Heart failure	1.32	1.22–1.43	1.09	1.00–1.18	1.22	1.16–1.28
Myocardial infarction	1.20	1.12–1.27	1.06	1.00–1.13	1.13	1.08–1.18
Mild liver disease	1.32	1.08–1.62	1.05	0.86–1.30	1.25	1.08–1.46
Peptic ulcer disease	1.22	1.15–1.30	1.16	1.09–1.23	1.06	1.01–1.10
Peripheral vascular disease	1.15	1.09–1.22	1.02	0.96–1.08	1.13	1.08–1.18
Renal disease	1.13	1.08–1.18	1.06	1.01–1.10	1.07	1.04–1.10
Stroke	1.25	1.15–1.35	1.06	0.98–1.15	1.18	1.11–1.24
Anxiety	1.08	1.03–1.12	1.03	0.99–1.08	1.04	1.01–1.08
Asthma	1.07	1.03–1.10	1.04	1.01–1.07	1.03	1.00–1.05
Depression	1.19	1.13–1.25	1.08	1.02–1.13	1.10	1.06–1.14
Contacts with GP in previous 12 months						
Low: 0–6	Referent					
Medium: 7–14	0.98	0.94–1.02	1.01	0.97–1.06	0.97	0.94–1.00
High: ≥15	1.27	1.21–1.34	1.14	1.08–1.19	1.12	1.08–1.16
COPD exacerbations in previous 12 months						
≥1 moderate-to-severe episode	1.44	1.37–1.51	1.13	1.07–1.18	1.28	1.24–1.32
≥1 severe episode	1.14	1.08–1.21	0.99	0.93–1.05	1.16	1.11–1.21

^a^Other smoker: no record about smoking history or record of passive smoking.

Abbreviations: CI, confidence interval; COPD, chronic obstructive pulmonary disease; GOLD, global initiative for obstructive lung disease; GP, general practitioner; MRC, Medical Research Council.

Determinants of dyspnoea grade were further assessed in models split by stage of airflow limitation. Associations similar to the main model were observed; prior history of COPD exacerbations was associated with increased dyspnoea grade, including mild dyspnoea, in each stage of airflow limitation (data not shown).

Table 5 shows frequency and rate of exacerbations during the 12 months’ follow-up by dyspnoea grade. Exacerbation rate per person-year increased with increasing level of dyspnoea (p<0.001). Similarly, at least one moderate-to-severe exacerbation was identified in 33% patients with MRC = 1 (no dyspnea), increasing to 67% for patients with MRC = 5. Similarly, for severe exacerbations, at least one event occurred in 7% of patients with MRC = 1 (no dyspnoea), increasing to 24% in patients with MRC = 5.

**Table 5:** Frequency of exacerbation events during the 12-month study period (from first recorded MRC dyspnoea grade from study start).

MRC dyspnoea grade	Cohort n	Events per patient,mean	Events per patient with ≥1event, mean	N (%) patientswith ≥1 event	p-value^a^
**Moderate-to-severe exacerbations**					
MRC = 1	6791	0.51	1.55	2244 (33.0)	Referent
MRC = 2	14,546	0.69	1.69	5955 (40.9)	<0.001
MRC = 3	10,036	0.94	1.87	5035 (50.2)	<0.001
MRC = 4	5689	1.28	2.17	3353 (58.9)	<0.001
MRC = 5	1194	1.58	2.37	795 (66.6)	<0.001
**Severe exacerbations**					
MRC = 1	6791	0.08	1.16	491 (7.2)	Referent
MRC = 2	14,546	0.11	1.14	1391 (9.6)	<0.001
MRC = 3	10,036	0.17	1.20	1415 (14.1)	<0.001
MRC = 4	5689	0.23	1.25	1061 (18.7)	<0.001
MRC = 5	1194	0.33	1.37	285 (23.9)	<0.001

^a^p-value estimated, using negative binomial regression, for a distribution of exacerbations between patients with a specific grade (MRC = 1, no dyspnoea is a referent category).

Abbreviation: MRC, Medical Research Council.

In the multivariate model (Table 6), dyspnoea grade was significantly associated with moderate-to-severe exacerbation rate during follow-up after controlling for demographic and clinical characteristics, producing increased risks of 18% for mild dyspnoea (MRC = 2: OR, 95% CI: 1.18, 1.13–1.23) and 46% for MRC≥3 (moderate-to-severe dyspnoea: OR, 95% CI: 1.46, 1.40–1.52), compared with MRC = 1 (no dyspnoea).

**Table 6:** Factors associated with risk of moderate-to-severe exacerbation during the 12-months’ follow-up (negative binomial regression).

Factor	Odds ratio	95% CI	p-value
Dyspnoea Grade			
MRC Grade 1	Referent		
MRC Grade 2	1.18	1.13–1.23	<0.0001
MRC Grade ≥3	1.46	1.40–1.52	<0.0001
Sex			
Female	Referent		
Male	0.92	0.89–0.94	<0.0001
Smoking status			
Current smoker	Referent		
Ex-smoker	0.98	0.95–1.01	0.2247
Non-smoker	0.85	0.81–0.89	<0.0001
Other	0.95	0.89–1.02	0.166
COPD GOLD stage nearest to the dyspnoea index date			
Stage I (FEV_1_≥80% pred.)	Referent		
Stage II (≥50% FEV_1_<80% pred.)	1.13	1.09–1.18	<0.0001
Stage III (≥30% FEV_1_<50% pred.)	1.43	1.37–1.50	<0.0001
Stage IV (FEV_1_>30% pred.)	1.73	1.63–1.84	<0.0001
Comorbidities anytime in their history prior to dyspnoea index date^a^			
Cancer	0.99	0.99–1.00	0.0227
Diabetes	1.01	1.00–1.01	0.004
Anxiety	0.99	0.98–0.99	<0.0001
Asthma	0.98	0.97–0.98	<0.0001
Depression	0.99	0.99–1.00	0.0002
Exacerbations of COPD^a^			
Any COPD exacerbation in previous 12 months	1.13	1.13–1.14	<0.0001
Contacts with GP in previous 12 months			
Low: 0–6	Referent		
Medium: 7–14	1.24	1.20–1.29	<0.0001
High: ≥15	1.43	1.38–1.49	<0.0001

^a^Referent group always constitutes patients without the trait anytime in their history prior to dyspnoea index date.

Abbreviations: CI, confidence interval; COPD, chronic obstructive pulmonary disease; GOLD, global initiative for obstructive lung disease; GP, general practitioner; MRC, Medical Research Council; QOF, UK National Health Service Quality and Outcomes Framework.

## Discussion

The Quality Outcomes Framework (QOF) introduced recording of the MRC dyspnoea grade as an indicator of care for patients with COPD in the NHS (England and Wales) in April 2009 [9]. Our COPD cohort of about 50,000 patients with COPD, selected from a general practice database representative of the UK, shows a rapid uptake of recording of dyspnoea in the study period starting on April 1, 2009, with about 82% of patients with diagnosed COPD having at least one record of MRC grade recorded.

Dyspnoea was recorded for the majority of the patients with about 40% reporting moderate or severe dyspnoea (MRC≥3, equal to mMRC≥2). This finding corresponds with results from observational studies conducted in Europe and the US, involving selective populations of COPD patients recruited from primary care, that the majority of patients reported at least mild perception of dyspnoea [8], [9]. Dyspnoea in COPD patients is excessive compared with the general population, in which moderate-to-severe dyspnoea has been reported to be 6% in people aged 20–65 years [14] and about 30% in those aged 65 years and older [27].

Increasing levels of dyspnoea were associated with higher disease severity and increased risk of poor outcomes. Patients reporting mild dyspnoea (MRC = 2, equal to mMRC 1) were distinct from patients reporting no dyspnoea (MRC = 1, equal to mMRC 0) or moderate-to- severe dyspnoea (MRC≥3) on both the association with risk factors and with outcomes. Even a mild grade of dyspnoea was an independent marker of an increased risk of moderate-to-severe exacerbations, a finding that is, to our knowledge, a new observation. The large COPD cohort size enabled us to discriminate patients’ characteristics and outcomes between pre-specified levels of absence of dyspnoea, mild dyspnoea, and moderate-to-severe dyspnoea. Bestall and colleagues coined the term ‘clinically significant dyspnoea’ for MRC≥3 [2]. They established dyspnoea as a cardinal symptom of COPD and described a relationship between increasing dyspnoea grade and COPD characteristics, although their study did not evaluate patients with no or mild dyspnoea (MRC = 1 or 2). Past studies have often merged mMRC grades 0 and 1 (equal to MRC grades 1 and 2) and labelled them as ‘no dyspnoea’. Our study, aided by its size and focus on pre-defined cut points of mild and moderate-to-severe dyspnoea, has demonstrated a distinct category of patients reporting mild dyspnoea on exertion (MRC = 2).

Dyspnoea was highly prevalent across all stages of airflow limitation in our study cohort. This observation is in agreement with previously published observational studies showing a consistent, though not very strong, relationship between dyspnoea and airflow limitation [9], [28]. Furthermore, it was shown that the progression of dyspnoea was dissociated from changes in FEV_1_, indicating the complex relationship between airflow limitation and dyspnoea [13], [29]. However, it is worth noting that dyspnoea itself is a complex and highly subjective trait, as described in a number of studies. Dynamic hyperinflation had been shown to contribute to the perception of exertional dyspnoea [30] and extensive disruptions in pulmonary gas exchange and small airway dysfunction leading into dynamic hyperinflation have been associated with exercise-related dyspnoea in mild airway obstruction (by FEV_1_ criteria [31]). Dynamic hyperinflation parameters, e.g. total lung capacity (TLC) and inspiratory capacity (IC), were shown to have a strong relationship with exercise-related dyspnoea [27], [31], [32]. Finally, acute administration of inhaled bronchodilators leads, among else, to reduction of dynamic hyperinflation, which has been linked to significant improvements in exercise-related dyspnoea [33].

The CPRD population source database used in this study contains records of FVC, which can indirectly approximate lung hyperventilation [34], but only in a subset of patients (N∼20,520), representing about a half of the total cohort. Therefore, we did not include FVC in the main analysis. When evaluating a relationship of dyspnoea grade and FVC in this sub-sample we observed a moderate correlation (Pearson r = –0.303), which corresponds with prior observations of O’Donnell and colleagues of a weak association of exercise tolerance and FVC [35]. As indirect, spirometrically derived assessments of lung hyperinflation do not allow the presence of a concomitant restrictive ventilatory deficit to be excluded, the inclusion of FVC may not enhance our results substantially. Finally, although the overall airflow limitation relationship with dyspnoea was only moderate in the multivariate analysis, more advanced stages of airflow limitation (FEV_1_<50% predicted) were the most significant determinant of higher dyspnoea grades, findings which also reflect the dissociation between dyspnoea and severity of COPD as assessed spirometrically (based on FEV_1_).

**Figure 5:**
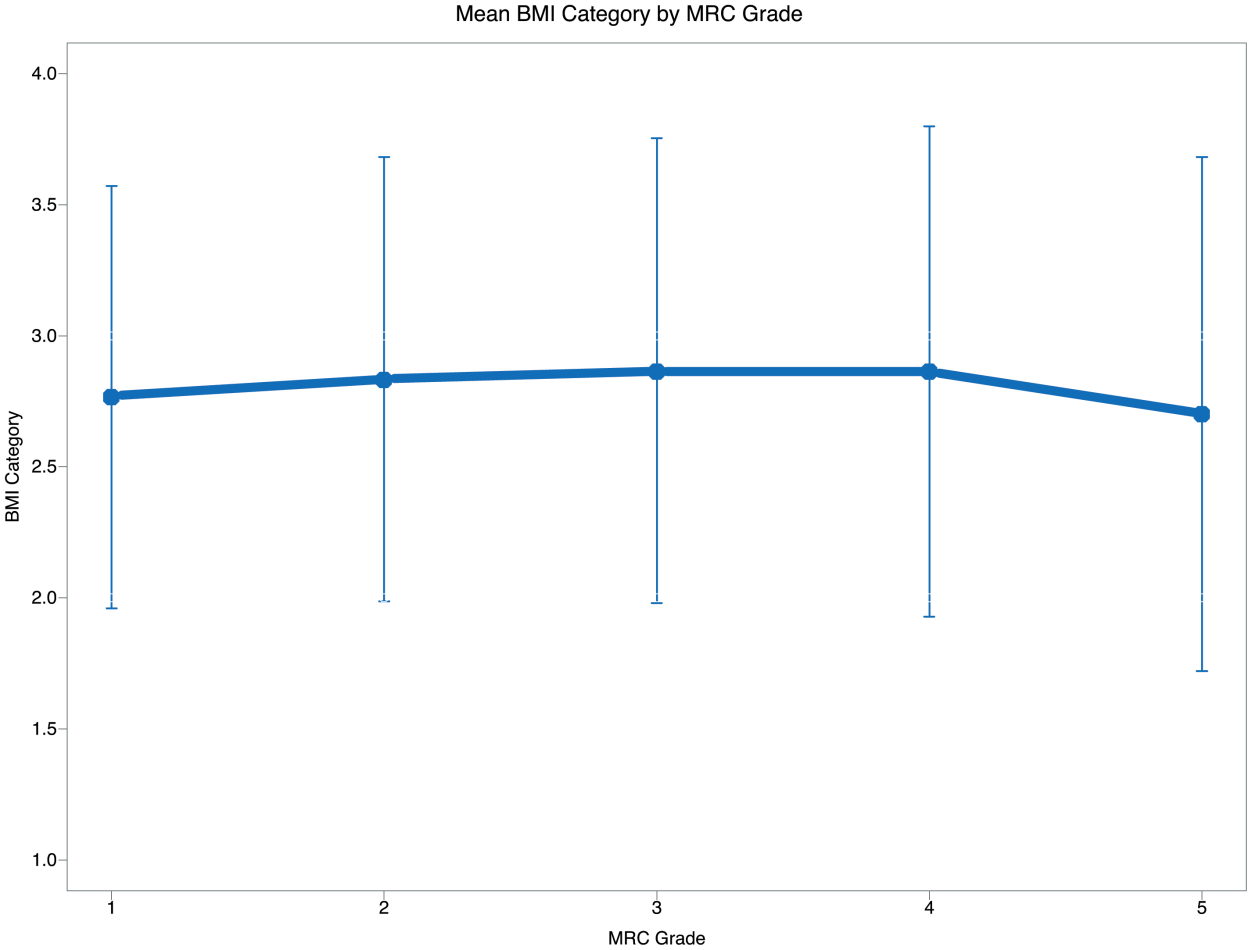
Line plot of mean BMI and MRC grade. Abbreviations: BMI, body mass index; MRC, Medical Research Council. MRC scoring 1–5 equals to mMRC grades 0–4 with MRC 1 equals to mMRC 0.

More severe dyspnoea was reported by females, smokers, the elderly, the obese, and those with a diagnosis of heart failure and past history of COPD exacerbations. The majority of these findings have been already communicated in previous reports [27], [36]. The relationship between dyspnoea and BMI resembled a U-shaped curve with underweight, normal weight, and obese patients reporting more dyspnoea than overweight patients (Table 4, fig. 5). There is an anecdotic evidence of underweight being associated with exercise related dyspnoea in COPD patients [37]. Reduced carbon monoxide diffusing capacity (DCO) and respiratory muscle strength are, at least in part, responsible for the enhanced sensation of dyspnoea in underweight emphysematous patients [37].

It is important to consider whether comorbid cardiovascular conditions can also contribute to dyspnoea in patients with COPD, highlighting the need for comprehensive management of chronic diseases in this population [38]. Dyspnoea has not only been associated with COPD, but is also one of the major symptoms of cardiac diseases. Indeed, we observed that the presence of heart failure was associated with an increased risk of dyspnoea. We also demonstrated a relatively small, but consistent increase in dyspnoea risk associated with most other comorbid diseases in the analysis.

Dyspnoea, both moderate-to-severe (MRC≥3) and mild (MRC = 2), was an independent predictor of exacerbation frequency during the 12-month observation period. The association between increased dyspnoea in patients reporting COPD exacerbations can be explained, at least in part, by the acceleration of disease progression associated with repeated exacerbations [39]; [40]. Despite indication of dyspnoea being an independent predictor of future exacerbations, it is also possible that the strongest predictor, which was the past history of exacerbations could have determined the relationship between dyspnoea and prospective exacerbations. Another possible mechanism can be related to increased hyperinflation and gas trapping, with reduced expiratory flow during exacerbations, leading to increased perception of dyspnoea [1], [30].

The strengths of this study include its size and the representativeness of the cohort to the diagnosed and managed COPD population in the UK. We identified almost 50,000 patients with well-characterized disease in primary care with a wide spectrum of disease severity as defined by airflow limitation, exacerbation frequency (or lack of exacerbations) and dyspnoea grade. Assessment of dyspnoea and spirometry were conducted as part of routine care.

Potential limitations of this study include the research focus of the database. As CPRD is a research database, GPs are aware that their data contribute to medical research. This influences both clinical behaviour and record management, and may not represent the general patient population treated by GPs who are not as motivated to participate in research. We also do not know if general practices compliant with QOF, measuring both FEV_1_ and MRC grade, are selecting participating patients in any way, e.g. according to disease severity. Further, we only selected patients with a recent diagnosis of COPD and with lung function measurements. Thus, our population may be skewed to those patients whose disease is actively managed. By applying this limitation, however, we were able to evaluate a relationship between dyspnoea and airflow limitation. In addition, the act of recording COPD diagnosis, dyspnoea and FEV_1_ can be a source of limitation as little is known about the quality of recording. The COPD diagnosis medical codes were validated in the study by Soriano and colleagues [41], but some codes have since been updated, reflecting the changes in the CPRD dictionary. Concerns about validity of spirometry conduct and utilization in patient care were recently raised in a study conducted in England [42]. The recording of dyspnoea severity, which is also a recommended component of a routine patient annual assessment, also relies on the quality of administration of the instrument and recording in a primary care setting. In addition, we cannot exclude the possibility that some patients may have their MRC grade impacted by a recent exacerbation. We did not measure a time from the last exacerbation to the dyspnoea code record used in this analysis because the exact timing of the start and end of exacerbation episodes have been modelled based on a priori assumptions, rather than observed and recorded in the database.

In conclusion, dyspnoea on exertion is commonly reported by patients across all levels of airflow limitation. The presence of dyspnoea in patients with COPD was associated with markers of greater disease severity and increased risk of poor outcomes.
